# Flavonoid Glycosides of *Polygonum capitatum* Protect against Inflammation Associated with *Helicobacter pylori* Infection

**DOI:** 10.1371/journal.pone.0126584

**Published:** 2015-05-18

**Authors:** Shu Zhang, Fei Mo, Zhaoxun Luo, Jian Huang, Chaoqin Sun, Ran Zhang

**Affiliations:** 1 Guiyang medical college, Guiyang, 550004, China; 2 Affiliated hospital of Guiyang Medical College, Guiyang, 550004, China; 3 Liaocheng people’s hospital, Shandong, 252000, China; Indian Institute of Science, INDIA

## Abstract

The antibacterial and anti-inflammatory activities, and protective effects of extracts (flavonoid glycosides) of *Polygonum capitatum* were investigated to detect the evidence for the utilization of the herb in the clinical therapy of gastritis caused by *H*. *pylori*. A mouse gastritis model was established using *H*. *pylori*. According to treating methods, model mice were random assigned into a model group (MG group), a triple antibiotics group (TG group, clarithromycin, omeprazole and amoxicillin), low/middle/high concentrations of flavonoid glycosides groups (LF, MF and HF groups) and low/middle/high concentrations of flavonoid glycosides and amoxicillin groups (LFA, MFA and HFA groups). A group with pathogen-free mice was regarded as a control group (CG group). The eradicate rates of *H*. *pylori* were 100%, 93%, 89% in TG, MFA and HF groups. The serum levels of IFN-gamma and gastrin were higher in a MG group than those from all other groups (*P* < 0.05). The serum levels of IFN-gamma and gastrin were reduced significantly in LF, MF and HF groups (*P* < 0.05) while little changes were observed in LFA, MFA and HFA groups. In contrast, the serum levels of IL-4 were lower and higher in MG and CG groups compared with other groups (*P<0*.*05*). The serum levels of IL-4 were increased significantly in LF, MF and HF groups (*P* < 0.05) while little changes were found in LFA, MFA and HFA groups. According to pathological scores, flavonoid glycosides therapy showed better protection for gastric injuries than the combination of flavonoid glycoside and amoxicillin (*P < 0*.*05*). The results suggested that flavonoid glycoside has repairing functions for gastric injuries. The results suggest that the plant can treat gastritis and protect against gastric injuries. The flavonoid glycosides from *Polygonum capitatum* should be developed as a potential drug for the therapy of gastritis caused by *H*. *pylori*.

## Introduction

*Helicobacter pylori (H*. *pylori)* infection is the main cause of chronic inflammation (gastritis), which is the second leading cause of gastric cancer in the world [1]. *H*. *pylori* infection also causes gastrointestinal lymphoma, which develop in stomach[2]. Mucosa-associated lymphoid tissue (MALT) and diffusing large B-cell lymphoma are the common histologic characters of gastric lymphoma[3]. Additionally, *H*. *pylori* infection is closely associated with the development of MALT lymphoma, which also results in gastric cancer[4]. The occurrence of selected genes such as gastrin and somatostatin[5], determine the pathogenicity of *H*. *pylori*. These proteins are pathogens contributing to peptic inflammation, ulceration, and cancer [6–8]. *H*. *pylori* eradication can completely control the development of MALT lymphomas[9] and has become the main focus for the prevention of gastric disease.

Presently, metronidazole, clarithromycine and amoxicillin are mostly used medicine for the therapy of *H*. *pylori* [10, 11]. However, a high prevalence of medicine resistance has been widely reported in *H*. *pylori* and the mechanisms for causing medicine resistance are complex [12–14]. The rate for the eradication of *H*. *pylori* is even less than 50% in most places[15]. Amoxicillin is the most powerful medicine for the therapy of the bacteria, but the high prevalence of medicine resistance in *H*. *pylori* also limits its utilization [12, 16]. There is increasing evidence that amoxicillin causes severe adverse effects in most patients [17].

Traditional Chinese medicine (TCM) has been implied in the Chinese health care for more than two thousand years[18]. The side effects and adverse events of TCM are often regarded as generally mild and infrequent[19]. *Polygonum capitatum* (*P*. *capitatum*), a traditional Chinese Miao-nationality herb, has been widely used in the treatment of urologic diseases [20]. Recent pharmacological studies have demonstrated that the antibacterial and anti-inflammatory activities of *P*. *capitatum* can be used for treating urinary tract infections at a clinical stage [21]. Further work extends the application of *P*. *capitatum* in treating diseases caused by *H*. *pylori* (Chinese patent No. CN102824417A), but the molecular mechanism remains unknown.

In immune system, interferon (IFN)-gamma can stimulate macrophage release and plays a critical role in the immune response against infection and controlling intracellular pathogens [22, 23]. Aberrant IFN-gamma expression is linked to many autoinflammatory and autoimmune diseases. The importance of IFN-gamma in the immune system stems is mostly due to its immunostimulatory and immunomodulatory effects [24]. IFN-gamma is mainly produced by natural killer and natural killer T cells in immune response, and by cluster of differentiation CD4 Th1 and cytotoxic CD8 T cells when antigen-mediated immunity develops[25]. *H*. *pylori* infection is one of the major causes for gastroduodenal pathologies. The long-term persistence of bacterial infection and immune and inflammatory response will affect the levels of IFN-gamma. IFN-gamma promotes the severity of the induced gastric lesions during the host response to *H*. *pylori* [26].

The interleukin 4 (IL4) is a kind of cytokine inducing the differentiation of naive helper T cells to Th2 cells[27]. Upon activation by IL-4, Th2 cell produces additional IL-4 in a positive feedback way. The function of IL-4 is similar to that of Interleukin 13[28]. It has many biological roles, such as activating the proliferation of B cells and T cells. It plays an important role in humoral and adaptive immunity. IL-4 can induce B-cell class switching to the antibody IgE, and up-regulate the levels of MHC class II. IL-4 can decrease the levels of Th1 cells, macrophages, IFN-gamma, and dendritic cell IL-12. Elevated IL-4 is associated with inflammation and wound repair[29]. IL-4 can modulate macrophage activation and polarization into M2 and inhibit activation of macrophages into M1 cells[30]. An increase in M2 macrophages is accompanied by the production of IL-10 and TGF-β that result in a decrease of pathological inflammation[31].

Gastrin, a kind of peptide hormone, stimulates the production of gastric acid by the parietal cells from the stomach and promotes gastric motility. Gastrin is secreted by G cells from the pyloric antrum of the stomach[32]. Decreased levels of gastrin were found in healthy adults because of the change of *H*. *pylori* infection. The control of gastrin can prevent *H*. *pylori*-induced gastritis. Gastrin plays a critical role in the inflammatory reaction of the gastric mucosa caused by *H*. *pylori* infection [33]. Somatostatin, a kind of growth hormone-inhibiting hormone[34], is a peptide hormone regulating the endocrine system and affecting cell proliferation by interacting with G protein-coupled somatostatin receptors and inhibiting the release of secondary hormones. Somatostatin cell is an important regulator of gastric acid secretion and alteration in its numbers plays a key role in gastroduodenal disease. The alterations may correlate with the severity of inflammation and certain peptide-immune interactions in the gastric mucosa caused by *H*. *pylori* infection [35].

Flavonoid glycoside is one of main components of *P*. *capitatum* and shows anti-bacterial activities [21]. Furthermore, amoxicillin is often combined with other medicine to treat *H*. *pylori* related disease. Therefore, we want to know the effects of flavonoid glycoside, which were compared with effects of combination therapy of flavonoid glycoside and amoxicillin on *H*. *pylori* infected disease. Meanwhile, the levels of IFN-gamma, IL-4, Gastrin and Somatostatin were measured.

## Results

### Sensitivity test of *H*. *pylori* to flavonoid glycoside

**Fig 1:**
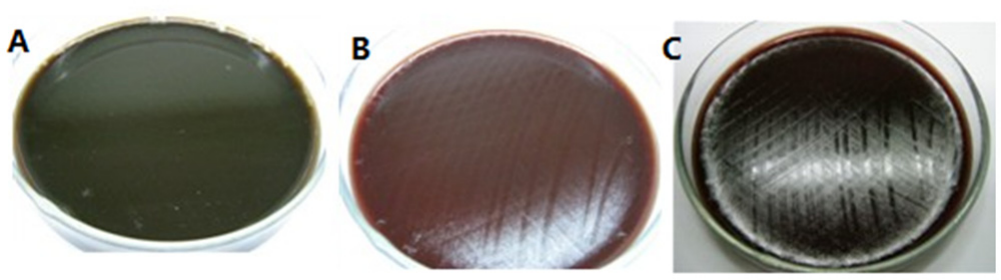
Sensitivity test of *H*. *pylori* to flavonoid glycoside. A: blank control. B: 40 ug/mL flavonoid glycoside. C: 80 ug/mL flavonoid glycoside.

In vitro antibacterial test, 40 ug/mL or higher concentrations of flavonoid glycoside inhibited the growth of *H*. *pylori* (Fig 1). Therefore, the MIC of flavonoid glycoside was 40 ug/mL, which was much higher than MIC of amoxicillin (1 ug/mL). The resistance of MIC of flavonoid glycoside was regarded as >40.0 μg/mL. The resistance of MIC of amoxicillin was regarded as >1.0 μg/ml.

### The toxicity of flavonoid glycoside on mice lymphocyte

High levels of flavonoid glycoside may inhibit the bioactivity of lymphocytes although flavonoid glycoside shows antibacterial activities [36]. There were not significant differences for mean OD values of lymphocytes when the concentrations were less than 64 ug/ml (*P* < 0.05). The OD values of lymphocytes were reduced significantly when the concentrations were more than 128 ug/ml (*P* < 0.05). The OD of lymphocytes reached the lowest level when the concentration of flavonoid glycoside was 512 ug/mL (Table 1).The results suggest that certain concentrations of flavonoid glycoside will not affect the proliferation of lymphocytes while the high concentrations of flavonoid glycoside will inhibit the proliferation of lymphocytes by its toxicity.

**Table 1:** CCK-8 colorimetric assay for detecting lymphocyte stimulation index after mice model treated with different concentrations of Flavonoid glycoside in vitro after 72 h.

Concentrations of Flavonoid glycoside	The OD of lymphocyte
**0 μ /mL**	0.673+0.0213
**1 μ /mL**	0.659+0.0138
**2 μ /mL**	0.712+0.0135
**4 μ /mL**	0.685+0.0165
**8 μ /mL**	0.598+0.0126
**16 μ6/mL**	0.647+0.0219
**32 μ/mL**	0.655+0.0240
**64 μ/mL**	0.542+0.0361
**128 μ/mL**	0.495+0.0271*
**256 μ/mL**	0.321±0.0230*
**512 μ/mL**	0.201±0.0172*
**1024 μ/mL**	0.225±0.0272*

Note:

*P<0.05 vs 0 ug/mL group.

### The establishment of a mouse model infected with *H*. *pylori*

**Fig 2:**
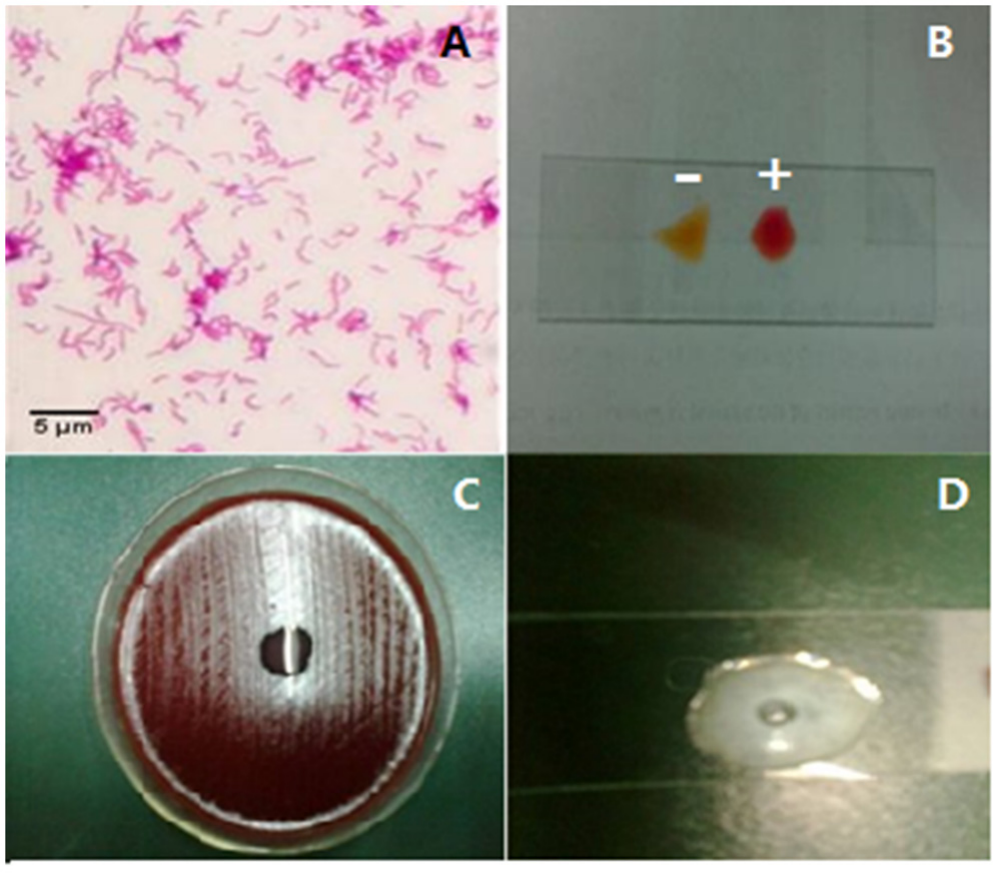
Identification of *H*. *pylori*. A: Gram staining, Gram staining differentiates bacteria by detecting peptidoglycan, which exists in the cell walls of gram-positive bacteria. After a Gram stain test, gram-positive bacteria shows the crystal violet dye, while a counterstain (safranin) added, gives gram-negative bacteria a pink coloring. B, rapid urease test, is a rapid diagnostic test for *H*. *pylori*. H. pylori secrete the urease enzyme, which can catalyze the conversion of urea to NH3 and raises the pH of the medium. The medium contains urea and an indicator such as phenol red, and thus the raised pH changes the color of the medium from yellow (a blank control) to red (an experimental group). C, the oxidase test is used to determine if a bacterium produces certain cytochrome c oxidases. It uses disks impregnated with a reagent such as TMPD, which is a redox indicator. The reagent can be changed from a dark-blue to maroon color when it is oxidized. D, the catalase test is one of main tests to identify *H*. *pylori*. The presence of catalase enzyme in the species is detected via hydrogen peroxide. If the bacteria possess catalase, when bacterial isolate is added to hydrogen peroxide, bubbles of oxygen will be observed. If the mixture produces bubbles, the organism is regarded as 'catalase-positive'.

The establishment of *H*. *pylori*-infected mice model was evaluated from two aspects: the identification of *H*. *pylori* isolated from gastric tissues of mice models and analysis of histochemistry and histopathology. *H*. *pylori* were identified via Gram staining, rapid urease test, oxidase test and catalase test. Gram staining differentiates bacteria by detecting peptidoglycan[37], which locates in the cell walls of gram-positive bacteria. After a Gram stain test, gram-positive bacteria showed the crystal violet dye, while a counterstain gave gram-negative bacteria a pink color (Fig 2A).Rapid urease test, is a rapid diagnostic test for *H*. *pylori*[38]. *H*. *pylori* secreted the urease enzyme, which catalyzed the conversion of urea to NH_3_ and raised the pH of the medium. The medium contained urea and an indicator phenol red, and thus the raised pH changed the color of the medium from yellow (a blank control) to red (an experimental group) (Fig 2B). The oxidase test was used to determine if a bacterium produced certain cytochrome c oxidases [39]. The disks were impregnated with a reagent such TMPD, which was a redox indicator. The reagent changed from dark-blue to maroon when it was oxidized (Fig 2C). The catalase test is one of main tests to identify *H*. *pylori*[40]. The presence of catalase enzyme in the species was detected via hydrogen peroxide. If the bacteria possessed catalase, bubbles of oxygen would be observed when bacterial isolate was added to hydrogen peroxide (Fig 2D).

**Fig 3:**
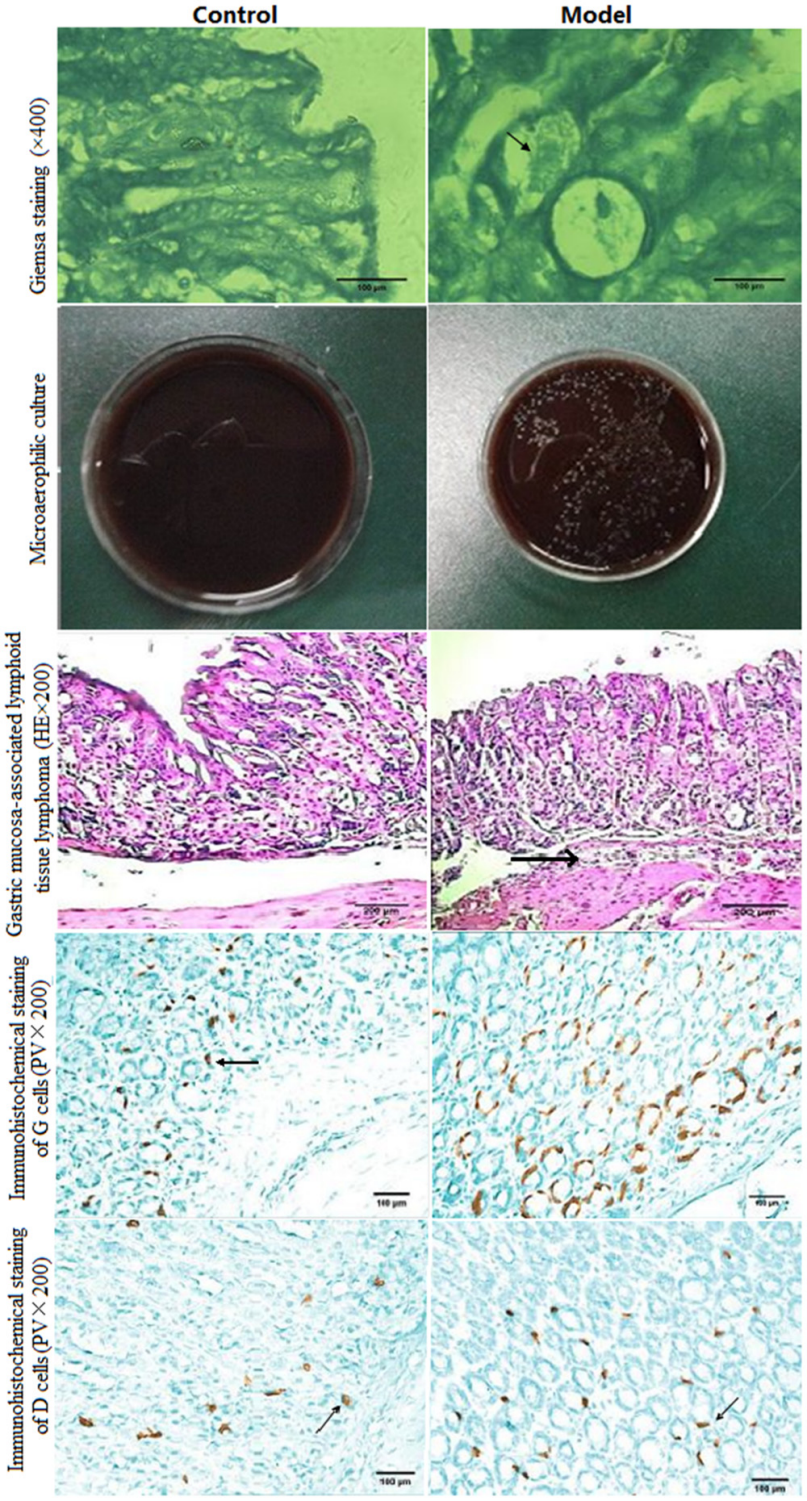
The comparision for histochemistry and histopathology between a control and a model group. Giemsa staining (×400), Giemsa stain was used to observe the adherence of pathogenic bacteria to gastric cells. Microaerophilic culturing of *H*. *pylori*, the extracellular *H*. *pylori* are microaerophilic. Hematoxylin and Eosin staining of gastric tissues was used for the detection of *H*. *pylori* (HE×200). The staining of G cells of Gastric mucosa-associated lymphoid tissue (MALT) lymphoma (PV×200). The staining of D cells of Gastric MALT lymphoma (PV×200).

Analysis of histochemistry and histopathology was performed via Giemsa staining, HE staining of mice gastric mucosa, microaerophilic culture and Immunohistochemical staining of G and D cells in Gastric mucosa-associated lymphoid tissue (MALT) lymphoma between control and model groups. Giemsa stain can be used to study the adherence of pathogenic bacteria to mammalians cells. In the control groups, no *H*. *pylori* were identified in the gastric mucosa. In contrast, *H*. *pylori* were observed in the gastric mucosa from mice models (Fig 3). Microaerophilic culture conditions are necessary for the growth of *H*. *pylori*[41]. Gastric biopsies were subjected to microaerophilic culture and then identified by the growth of *H*. *pylori* in mice models. In contrast, no colonies were observed in control groups (Fig 3). H&E staining was conducted to observe morphological alterations in gastric mucosa after *H*. *pylori* infection. Mucosal destruction in the gastric mucosa were observed in mice models. Representative inflammatory infiltrates were found at the base of the mucosa with lymphocytes and polymorphonuclears in the mice infected by *H*. *pylori*. In contrast, the mice showed normal gastric morphology without mucosal destruction and inflammatory infiltrates (Fig 3). Immunohistochemical staining of G cells in MALT lymphoma showed that the number of G cells in model groups was more that in a control group. Similarly, immunohistochemical staining of D cells in MALT lymphoma showed that the number of D cells in model groups was more that in a control group (Fig 3). All above information indicated that a *H*. *pylori*-infected model was successfully established.

### The effects of flavonoid glycoside on the eradication rate of *H*. *pylori*

Different groups showed different eradication rate of *H*. *pylori* (Table 2). Three kinds of antibiotics eradicated *H*. *pylori* completely in a TG group. The combination of flavonoid glycoside and amoxicillin eradicated *H*. *pylori* by more than 93% in a MFA group while only flavonoid glycoside eradicated *H*. *pylori* by 89% at most. According to the relative amounts of *H*. *pylori*-specific CagA gene determined by qRT-PCR[42], the log cells of per g of tissues were 0 in a TG group while the number is 6.6 ± 0.5 in a MG group. Comparatively, the numbers were 5.4 ± 0.3 in a MFA group and 5.8 ± 0.3 in a HF group. Flavonoid glycoside showed the similar antibacterial activities compared with the combination therapy of flavonoid glycoside and amoxicillin.

**Table 2:** Analysis of *H*. *pylori*clearance between model group and other treatment groups.

	Colonization density(Ig cfu/g)	*H*. *pylori* clearance	^a^Log cells of per g of wet tissues
**MG**	7.27±0.94	0	6.6 ± 0.5
**TG**	6.35 ± 0.78	100%	0
**LF**	6.31±0.89	76.66%	6.1 ± 0.4
**MF**	6.56±0.41	76.15%	6.1 ± 0.4
**HF**	6.64±0.42	89.07%	5.8 ± 0.3
**LFA**	6.15±1.03*	92.09%	5.5 ± 0.5
**MFA**	6.10±0.59*	93.32%	5.4 ± 0.3
**HFA**	6.17±0.85	92.57%	5.5 ± 0.4

Note:

^a^The number is determined by the relative amounts of *H*. *pylori*-specific CagA DNA.

*P<0.*05* vs model group (n = 8).

### Flavonoid glycoside improves the pathology of *H*. *pylori*-infected mice model

**Fig 4:**
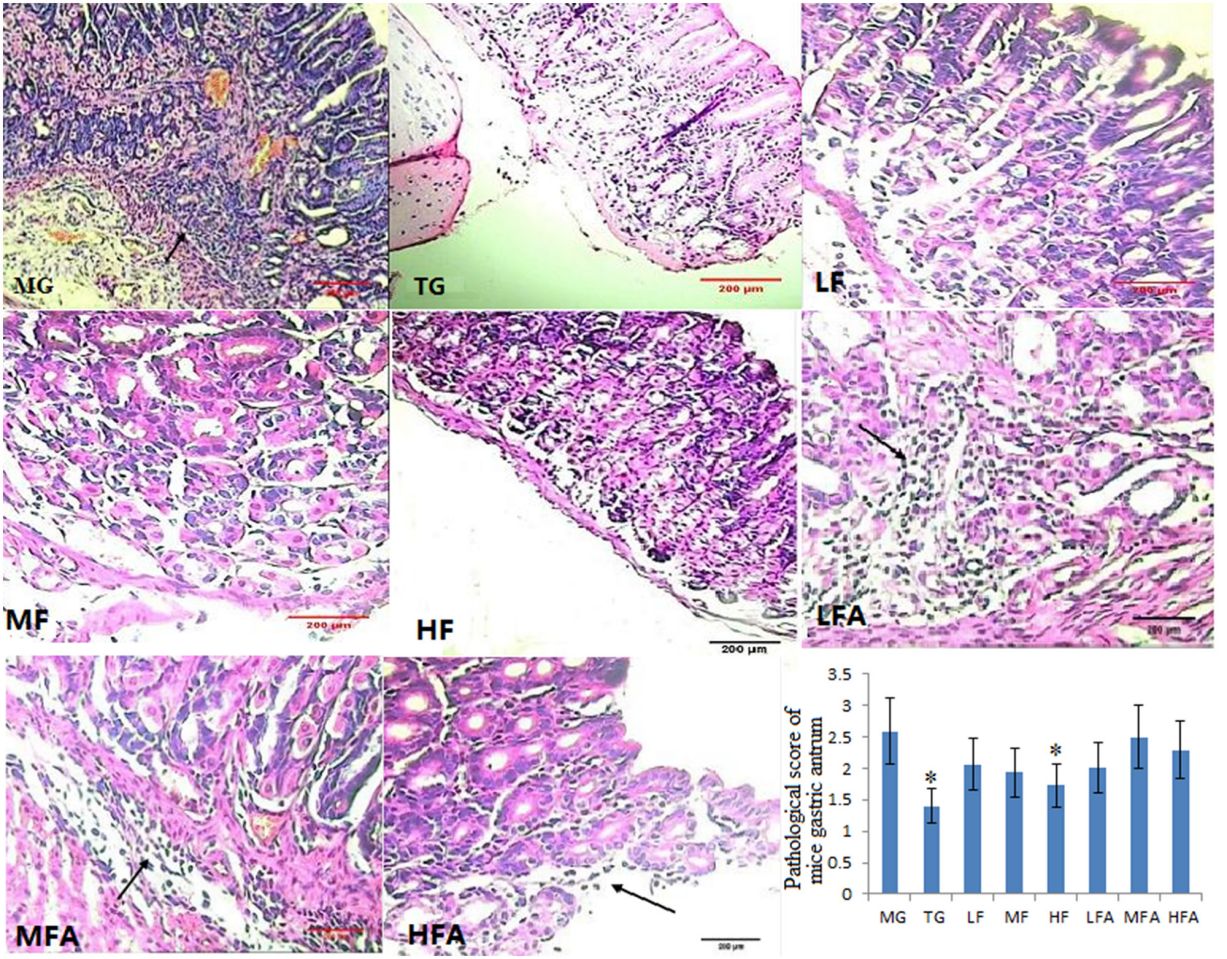
Hematoxylin and Eosin staining of gastric tissues (HE×200). Normal mice (without *H*. *pylori* infection) were assigned as a control group (CG group) and mice models infected with *H*. *pylori* were divided into model group (MG group, only treated with saline solution), triple combination therapy group (TG group, the daily medicine intake is 0.5 ug clarithromycin, 0.02 ug omeprazole, and 1 ug amoxicillin), low/middle/high concentrations of flavonoid glycoside group (LF/MF/HF group, treated with one daily dose of Flavonoid glycoside at 32/64/128 ug), low/middle/high concentrations of flavonoid glycoside and common concentration of amoxicillin group (LFA/MFA/HFA group, treated with one daily dose of flavonoid glycoside at 32/64/128 ug and amoxicillin at 1 ug). Pathological score of mice gastric antrum in each group (±SD, n = 10). *P < 0.05 vs model group.

We established a mouse gastritis model using *H*. *pylori* and the degree of infection was evaluated by H&E staining (Fig 4). In a CG group, gastric mucosa was found with a common morphology, and no inflammation was observed. These histopathological varies showed that a mouse model was established successfully. In a MG group, the structures of normal gastric mucosa were destroyed, and mucosal destruction was observed. The degree of infection was significantly alleviated and inflammatory cells (lymphocytes, monocytes, neutrophils) were reduced in TG and HF groups compared with those in MF, HF, LFA, MFA and HFA groups (Fig 4). In TG and HF groups, the gastric mucosa was in normal morphology and the pathological scores were also lower than other groups. In contrast, the gastric mucosa were destroyed with different degrees in MG, LF, MF, LFA, MFA and HFA groups, especially the pathological scores were the highest in a MG group. The results showed that flavonoid glycoside could improve the pathology of *H*. *pylori* infection significantly. More importantly, according to pathological scores, flavonoid glycoside showed better protect gastric tissues than the combination of flavonoid glycoside and amoxicillin (*P* < 0.05) (Fig 4). The results suggested that flavonoid glycoside has repairing functions for gastric injuries.

**Fig 5:**
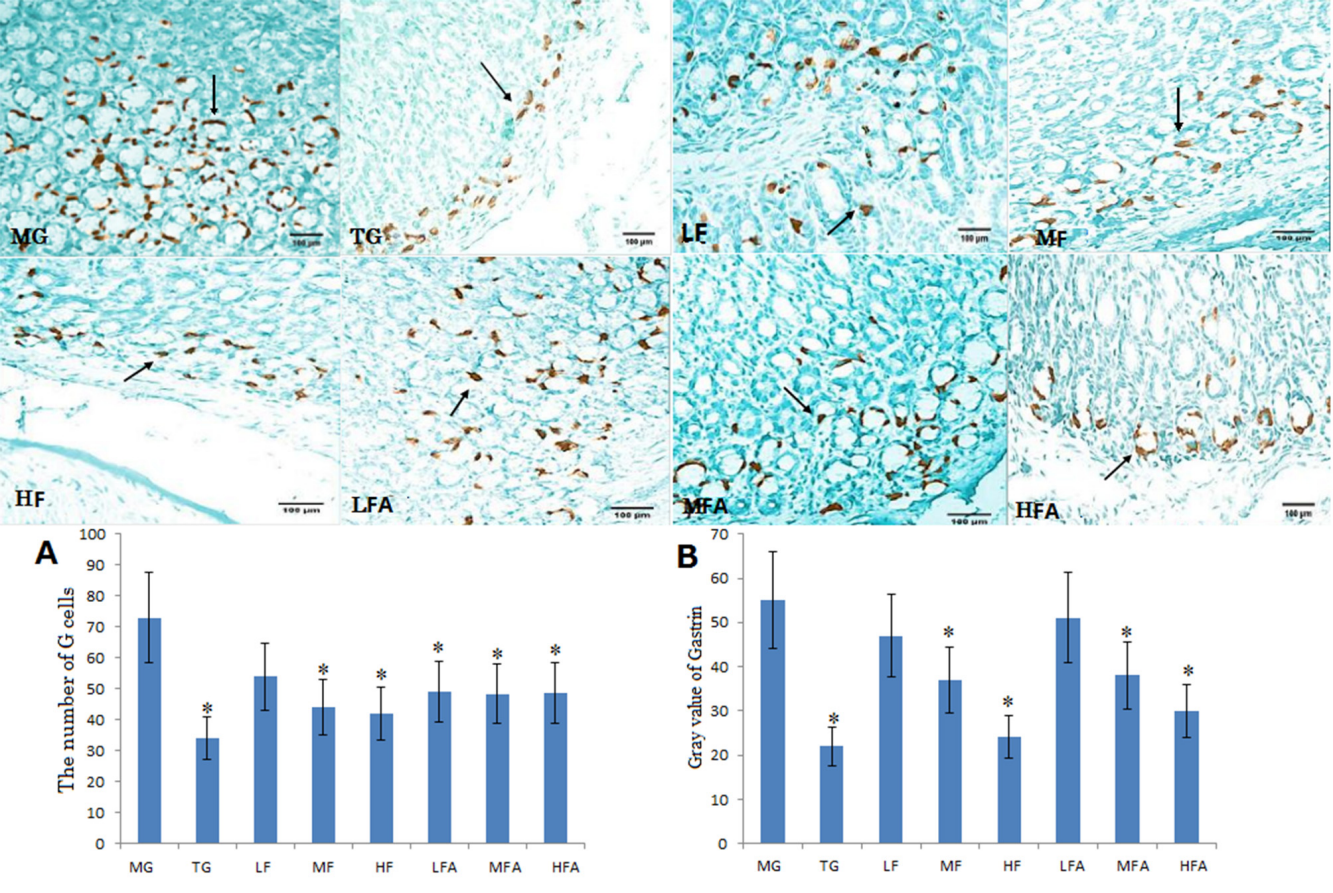
The staining of G cells of Gastric mucosa-associated lymphoid tissue (MALT) lymphoma (PV×200). Normal mice (without *H*. *pylori* infection) were assigned as a control group (CG group) and mice models infected with *H*. *pylori* were divided into model group (MG group, only treated with saline solution), triple combination therapy group (TG group, the daily medicine intake is 0.5 ug clarithromycin, 0.02 ug omeprazole, and 1 ug amoxicillin), low/middle/high concentrations of flavonoid glycoside group (LF/MF/HF group, treated with one daily dose of Flavonoid glycoside at 32/64/128 ug), low/middle/high concentrations of flavonoid glycoside and common concentration of amoxicillin group (LFA/MFA/HFA group, treated with one daily dose of Flavonoid glycoside at 32/64/128 ug and amoxicillin at 1 ug). Comparison of G cells and gastrin gray value in each group (±s, n = 10). *P < 0.05 vs model group.

Immunohistochemical staining of G cells MALT lymphoma was restricted to gastric cells in MG and LF groups, and a stronger immune staining was observed in the two groups. In contrast, G cells were weaker immune staining with a little light brown color in TG, MF, HF, LFA, MFA and HFA groups (Fig 5). A G cell staining was positively related with the degrees of *H*. *pylori* infection. Thus, the infection could be controlled well in TG, MF, HF, LFA, MFA and HFA groups. The results suggested that certain concentrations of flavonoid glycoside controlled the *H*. *pylori* infection well.

**Fig 6:**
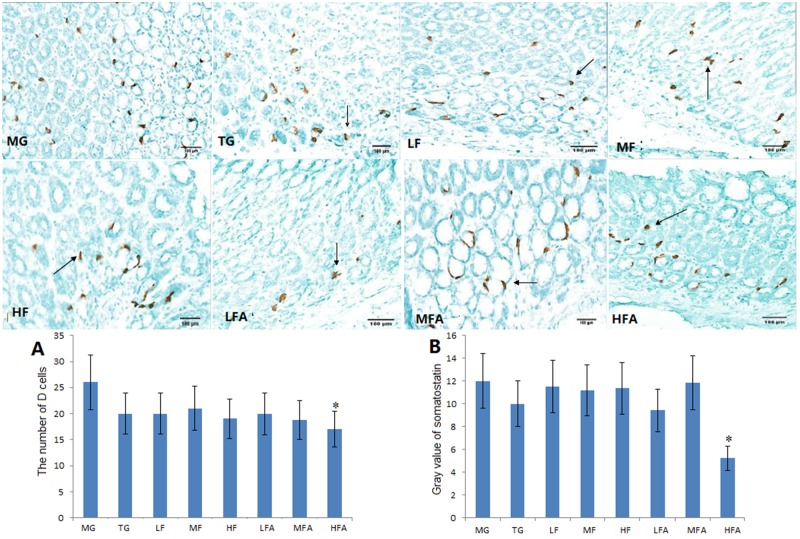
The staining of D cells of Gastric mucosa-associated lymphoid tissue (MALT) lymphoma (PV×200). Normal mice (without *H*. *pylori* infection) were assigned as a control group (CG group) and mice models infected with *H*. *pylori* were divided into model group (MG group, only treated with saline solution), triple combination therapy group (TG group, the daily medicine intake is 0.5 ug clarithromycin, 0.02 ug omeprazole, and 1 ug amoxicillin), low/middle/high concentrations of flavonoid glycoside group (LF/MF/HF group, treated with one daily dose of flavonoid glycoside at 32/64/128 ug), low/middle/high concentrations of flavonoid glycoside and common concentration of amoxicillin group (LFA/MFA/HFA group, treated with one daily dose of Flavonoid glycoside at 32/64/128 ug and amoxicillin at 1 ug). Analysis of G cells and somatostatin gray value in each group (n = 10). *P < 0.05 vs model group.

Comparably, immunohistochemical staining of D cells MALT lymphoma was restricted to gastric cells in MG, TG, LF, MF, HF, LFA and MFA groups, and a stronger immune staining was observed in these groups. In contrast, D cells were weaker immune staining with a little light brown color in a HFA group (Fig 6). All the results suggested that the combination therapy of high concentration of flavonoid glycoside and amoxicillin affected the number of D cells significantly, which was different from the effect on G cells.

### Effects of flavonoid glycoside and amoxicillin on the mRNA levels of IFN-gamma, IL-4, Gastrin and Somatostatin

We investigated the effects of flavonoid glycoside and amoxicillin on the mRNA levels of IFN-gamma, IL-4, gastrin and somatostatin, which can reflect the degrees of *H*. *pylori* infection [33, 43–45]. The mRNA levels of IFN-gamma were significantly higher in a MG group than those in all other groups (P < 0.05) (Table 3). The mRNA levels of IFN-gamma were significantly lower in CG, TG, HF and MF groups than those in all other groups (*P* < 0.05) (Table 4). All the results suggested that flavonoid glycoside affected the mRNA levels of IFN-gamma. In contrast, the mRNA levels of IL-4 were significantly lower in a MG group than those in all other groups (*P* < 0.05) (Table 4). The mRNA levels of IL-4 were significantly increased in CG, TG, HF and MF groups than those in all other groups (*P* < 0.05) (Table 4). All the results suggested that the treatment of flavonoid glycoside increased the mRNA levels of IL-4. Gastrin (GAS) is produced by G cells in mice stomach lining and associated with the number of G cells. Real-time qRT-PCR analysis showed that the levels of GAS were higher in a MG group than all other groups (*P* < 0.05) (Table 5). Similarly, somatostain (SS) is produced by D cells in mice stomach and associated with the number of D cells. Real-time qRT-PCR analysis showed that the levels of SS were higher in a MG group than all other groups (*P* < 0.05) (Table 6).

**Table 3:** The mRNA levels of IFN-gamma in the gastric mucosa of *H*. *pylori* infected mice in different groups (n = 6).

Groups	β-actin	IFN-gamma CT value	2-△△CT
MG	32.831±0.428	19.523±2.410	1▲
CG	35.000±1.004	18.513±3.261	0.110*
TG	33.796±2.416	17.768±1.945	0.150*
HF	33.093±1.024	16.995±3.584	0.166*
MF	33.964±3.739	18.669±3.105	0.269*
LF	35.000±0.692	21.377±5.042	0.451
HFA	31.179±1.296	18.513±2.178	0.874
MFA	30.198±1.397	17.029±3.791	0.912

^▲^ *P* < 0.05 model group vs blank group;

*P < 0.05 vs model group.

**Table 4:** The mRNA levels of IL-4 in the gastric mucosa of *H*. *pylori* infected mice in different groups (*n* = 6).

Groups	β-actin CT values	IL-4 CT values	2^-ΔΔCT^
**MG**	32.501±0.138	17.283±1.383	1^▲^
**CG**	30.651±1.027	18.388±1.972	7.755*
**TG**	31.872±0.987	18.837±2.936	4.831*
**HF**	31.198±0.237	17.988±1.097	4.022*
**MF**	30.868±0.487	17.390±2.945	3.324*
**LF**	31.989±1.002	18.292±0.843	2.869*
**HFA**	33.946±0.948	18.387±1.075	1.257
**MFA**	32.202±0.739	17.639±2.382	1.354
**LFA**	35.662±1.024	21.377±2.261	1.669

Note:

^▲^ *P* < 0.05 via a CG group,

*P < 0.05 via a MG group.

**Table 5:** The mRNA levels of Gastrin in the gastric mucosa of *H*. *pylori* infected mice in different groups (n = 6).

Groups	β-actin	GAS(CT)	2^-ΔΔCT^	LOG(2^-ΔΔCT^)
**MG**	20.68±0.97	20.56±0.79	1	0
**CG**	19.79±0.02	26.51±0.13	0.0093±0.0001*	-2.03
**TG**	20.01±0.55	25.45±0.72	0.0225±0.0001*	-1.65
**HF**	20.28±0.14	25.38±0.29	0.0286±0.0006*	-1.54
**MF**	20.48±0.23	26.16±0.03	0.0192±0.0010*	-1.72
**LF**	20.79±0.38	26.96±0.36	0.0135±0.0015*	-1.87
**HFA**	20.52±0.33	25.29±0.07	0.0361±0.0053*	-1.44
**MFA**	20.07±0.08	20.54±0.22	0.7095±0.0199	-0.15

*P < 0.05 vs model group

**Table 6:** The mRNA levels of Somatostatinin the gastric mucosa of *H*. *pylori* infected mice in different groups (*n* = 6).

Groups	β-actin (CT)	SS (CT)	2-^ΔΔ^CT	LOG(2-^ΔΔ^CT)
MG	20.68±0.97	19.07±1.04	1	0
CG	19.79±0.02	20.92±0.02	0.150±0.013*	-0.82
TG	20.01±0.55	20.61±0.37	0.217±0.015*	-0.66
HF	20.28±0.14	20.40±0.05	0.303±0.004*	-0.52
MF	20.48±0.23	19.86±0.25	0.506±0.018*	-0.30
LF	20.79±0.38	21.27±0.38	0.236±0.012*	-0.63
HFA	20.52±0.33	20.38±0.43	0.362±0.005*	-0.44
MFA	20.07±0.08	19.05±0.20	0.556±0.064*	-0.25

*P < 0.05 vs model group

### Effects of flavonoid glycoside and amoxicillin on the protein levels of inflammatory biomarkers

**Fig 7:**
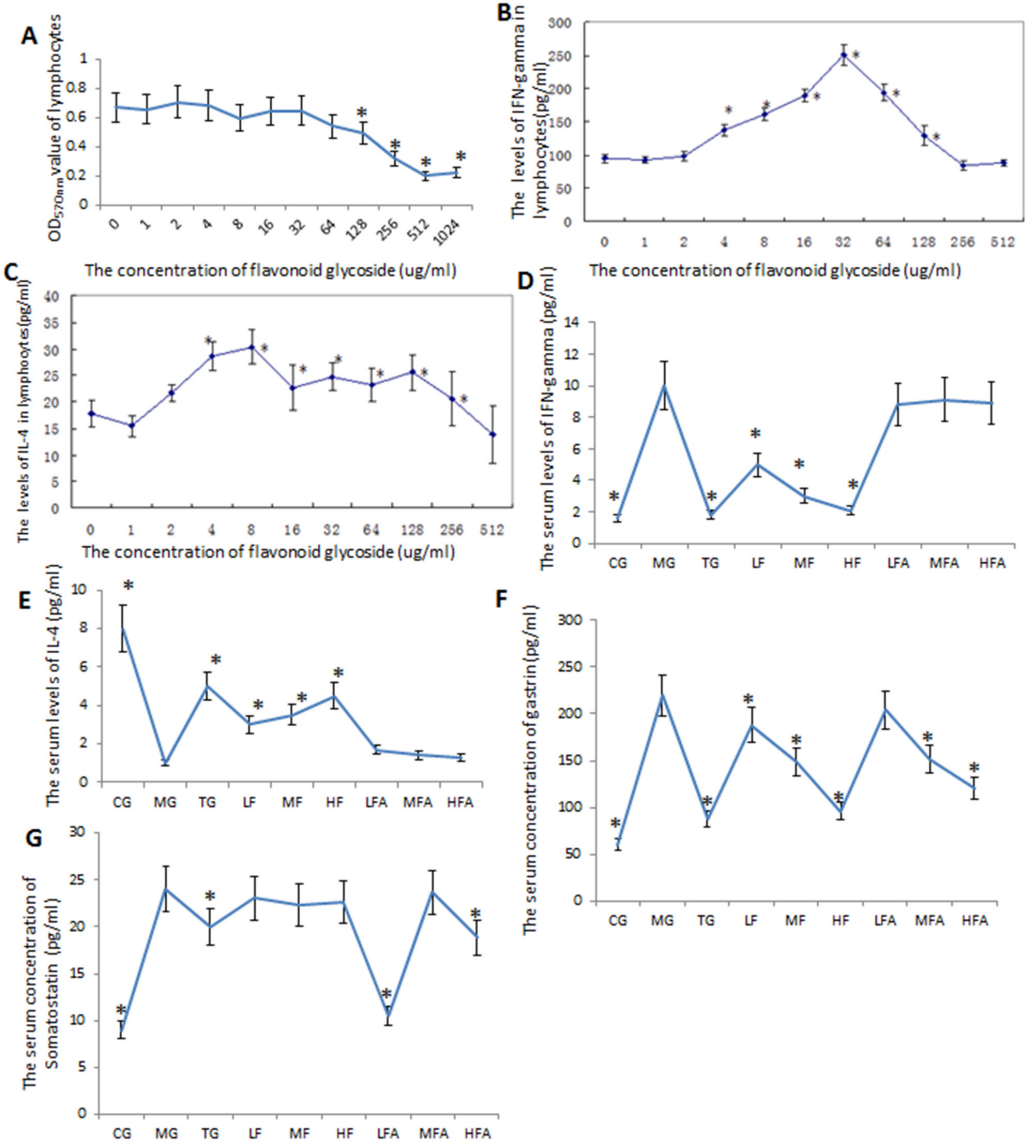
The effects of flavonoid glycoside on the number of lymphocytes, protein levels of inflammatory biomarkers. A, the effects of different concentrations of flavonoid glycoside on the number of lymphocytes. B, the effects of different concentrations of flavonoid glycoside on protein levels of INF-gamma. C, the effects of different concentrations of flavonoid glycoside on protein levels of IL-4. D, the serum levels of INF-gamma in different groups. E, the serum levels of IL-4 in different groups. F, the serum levels of gastrin in different groups. G, the serum levels of somatostatin in different groups. Normal mice (without *H*. *pylori* infection) were assigned as a control group (CG group) and mice models infected with *H*. *pylori* were divided into model group (MG group, only treated with saline solution), triple combination therapy group (TG group, the daily medicine intake is 0.5 ug clarithromycin, 0.02 ug omeprazole, and 1 ug amoxicillin), low/middle/high concentrations of flavonoid glycoside group (LF/MF/HF group, treated with one daily dose of flavonoid glycoside at 32/64/128 ug), low/middle/high concentrations of flavonoid glycoside and common concentration of amoxicillin group (LFA/MFA/HFA group, treated with one daily dose of flavonoid glycoside at 32/64/128 ug and amoxicillin at 1 ug). *P < 0.05 vs a model group (n = 10).

Before the study, the effects of flavonoid glycoside on lymphocytes were measured. The results showed that the OD values of lymphocytes were affected by the concentration of flavonoid glycoside. The OD values were the highest when no flavonoid glycoside was used and the values reached the lowest level when 512 ug/ml of flavonoid glycoside was used (*P* < 0.05) (Fig 7A). The levels of IFN-gamma from lymphocytes were also affected by flavonoid glycoside and the levels reached the highest point when 32 ug/ml of flavonoid glycoside was used (*P* < 0.05) (Fig 7B). Comparatively, the levels of IL-4 reached the highest point when 8 ug/ml of flavonoid glycoside was used (*P* < 0.05) (Fig 7C). To explore the effects of flavonoid glycoside and amoxicillin on IFN-gamma and IL-4, the serum levels of IFN-gamma and IL-4 were measured in different groups. The results indicated that the serum levels of IFN-gamma were higher in a MG group than those in all other groups (*P* < 0.05) (Fig 7D). The groups only with flavonoid glycoside could reduce the serum levels of IFN-gamma significantly (*P* < 0.05) while the combination of flavonoid glycoside and amoxicillin caused no obvious changes. In contrast, the serum levels of IL-4 were lower in a MG group than those from all other groups while the levels in a CG group were higher than those from all other groups (*P* < 0.05) (Fig 7E). The groups only with flavonoid glycoside could increase the serum levels of IL-4 significantly (*P* < 0.05) while the combination of flavonoid glycoside and amoxicillin caused no obvious changes. For gastrin, the serum levels were higher in a MG group than those in all other groups except a LF group (*P* < 0.05) (Fig 7F). The concentrations of serum gastrin reached the lowest level in HF, TG and CG groups. Comparatively, the serum levels of somatostatin were higher in all groups only with flavonoid glycoside, a MFA group and a MG group than those from other groups (*P* < 0.05) (Fig 7G). The concentrations of serum somatostatin reached the lowest level in LFA and CG groups.

### The effects of flavonoid glycosides on Gastric cells infected by *H*. *pylori*

**Fig 8:**
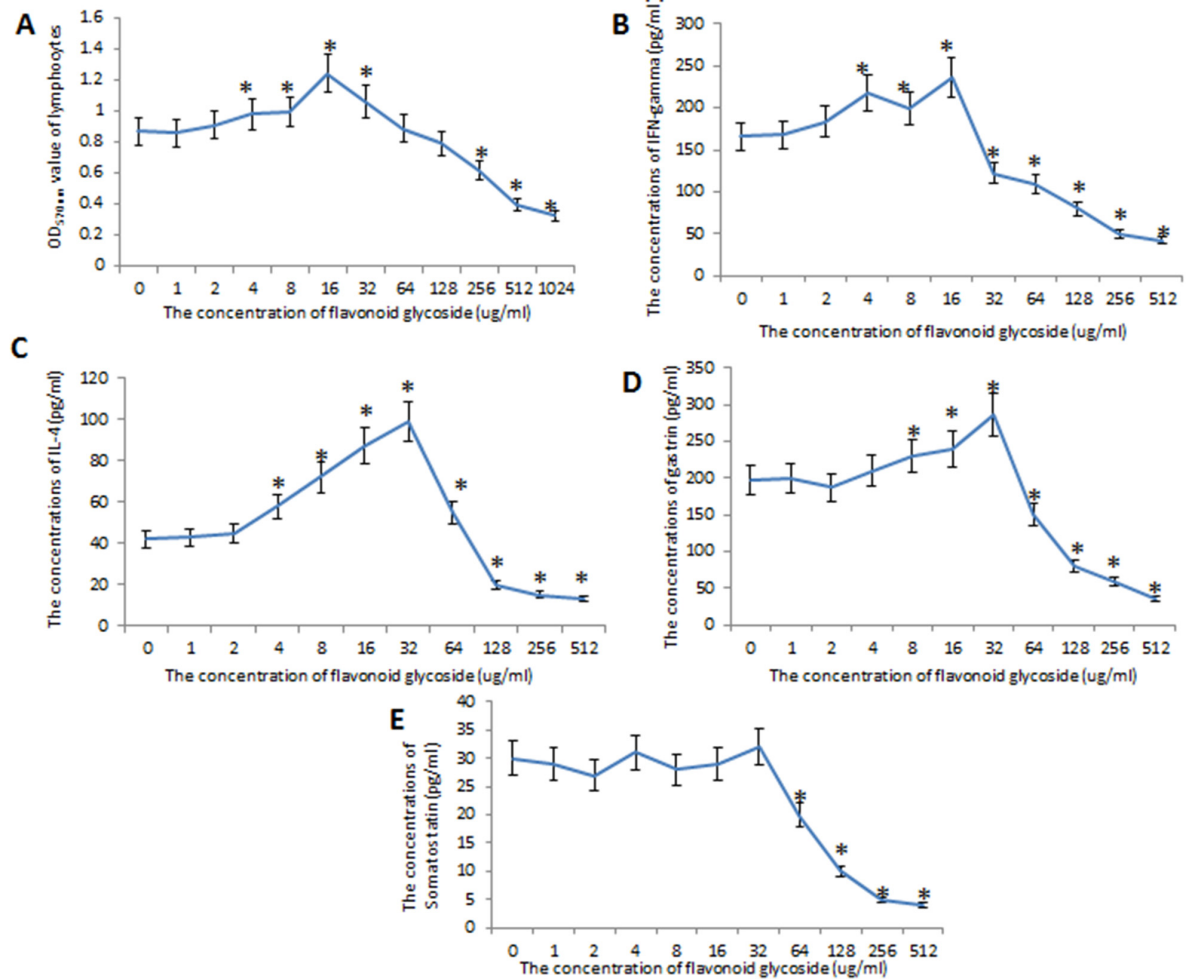
The effects of flavonoid glycoside on gastric cells infected by *H*. *pylori*. A, the effects of different concentrations of flavonoid glycoside on the number of gastric cells MGC803. B, the effects of different concentrations of flavonoid glycoside on protein levels of INF-gamma. C, the effects of different concentrations of flavonoid glycoside on protein levels of IL-4. D, the effects of different concentrations of flavonoid glycoside on protein levels of gastrin. E, the effects of different concentrations of flavonoid glycoside on protein levels of somatostatin. *P < 0.05 vs a control group without flavonoid glycoside.

Before the study, the effects of flavonoid glycoside on gastric cells were measured. The results showed that the OD values of gastric cells were affected by the concentrations of flavonoid glycoside. The OD values were the highest when 16 flavonoid glycoside was used and the values reached the lowest level when the concentrations were more than 512 ug/ml (*P* < 0.05) (Fig 8A). The levels of IFN-gamma from lymphocytes were also affected by flavonoid glycoside and the levels reached the highest point when 16 ug/ml of flavonoid glycoside was used (*P* < 0.05) (Fig 8B). Comparatively, the levels of IL-4 and gastrin reached the highest point when 32 ug/ml of flavonoid glycoside was used (*P* < 0.05) (Fig 8C and 8D). The levels of somatostatin were not affected by flavonoid glycoside if the concentrations were less than 32 ug/ml of flavonoid glycoside (*P* < 0.05) (Fig 8E). All these biomarkers would be reduced greatly if the concentrations were more than 64 ug/ml, suggesting that high concentrations of flavonoid glycoside have toxicity toward gastric cells and inhibit the production of these molecules. All these results implied that flavonoid glycoside may show its anti-inflammatory functions by increasing the levels of IFN-gamma, IL-4 and gastrin.

## Discussion

*H*. *pylori* infection is well known to be associated with the risk of many diseases. For example, *H*. *pylori* infection contributes to cardiovascular diseases and stroke, and Alzheimer's disease (AD) [46]. Another example, *H*. *pylori* infection is most likely to cause chronic gastritis, peptic ulcer disease and liver-related diseases[47]. *H*. *pylori* infection significantly affects the life quality of many people in the world and has become a global burden. *H*. *pylori* infection therapy often includes pharmaceutical treatment, such as clarithromycin, omeprazole and amoxicillin [48–50]. However, all the medicine has side effects which greatly limit the usage: Omeprazole treatment can cause hypergastrinemia and trophic effects in the stomach with an increase of histamine-producing enterochromaffin-like cells[51]; Combination therapy of clarithromycin and rabeprazole can increase the risk of neurotoxicity[52]; Amoxicillin therapy can also lead to severe adverse effects and death[17]. Thus, it is necessary to explore the new medicine with fewer side effects and therapeutic efficacy. Flavonoid glycoside is a kind of Chinese herbs and has been widely used for the therapy of urinary tract infection [21]. Recent work indicates that flavonoid glycoside can be used for the treatment of *H*. *pylori* infection with a few side effects (Chinese patent No. CN102824417A). Amoxicillin is often combined with other medicine for the therapy of H. pylori infection [53], so the combination therapy of flavonoid glycoside and amoxicillin is an effective way for the therapy of *H*. *pylori* infection.

To understand the effects of flavonoid glycoside and amoxicillin on a *H*. *pylori* infected disease, a mouse gastritis model was established using *H*. *pylori*. To explore the molecular mechanisms of effects of flavonoid glycoside and amoxicillin on a mouse gastritis model, IFN-gamma, IL-4, gastrin and somatostatin may be the best molecules for the purpose because of the following reasons: *H*. *pylori* infection can elevate IFN-gamma-mediated gastric inflammation [43]; IL-4 is an anti-inflammatory and Th2-type cytokine. *H*. *pylori* infection in mammals induces an immune response, which is characterized by an increase of IFN-gamma and absence of IL-4[54]; Serum levels of gastrin are higher in *H*. *pylori*-infected patients than in uninfected subjects, and *H*. *pylori* infection induces hypergastrinemia in mammals[55]; Somatostatin is a regulatory peptide, which is mainly existed in the stomach. Somatostatin is needed for IL-4-mediated resolution of *H*. *pylori* gastritis [56]. Flavonoid glycoside intervention can reduce the serum levels of INF-gamma but not IL-4(Figs 7D and 8E). However, the combination of flavonoid glycoside and amoxicillin doesn’t change the levels of INF-gamma, which cannot improve the inflammation of the tissues infected with *H*. *pylori*. The results suggested that only flavonoid glycoside therapy can control the inflammation caused by *H*. *pylori* infection.

For further mechanism, flavonoid glycoside has the potential antioxidant activity and the antioxidant functions of flavonoid glycoside have been reported[57]. Most species of *Polygonum* have bioactive constituents, which contribute to many medicinal properties. *P*. *cuspidatum* and *P*. *capitatum* exhibit great antioxidant properties and are a potent resource of natural bioactive antioxidants. Here, we found only flavonoid glycoside treatment repaired the gastric injury compared with those treated with the combination of flavonoid glycoside and amoxicillin. The difference may be caused by the antioxidant bioactivities of flavonoid glycoside and side effects of amoxicillin for gastric mucosa. Therefore, we explored the protect function of flavonoid glycoside for gastric mucosa. A high concentration of amoxicillin may be harmful to gastric mucosa although it can enhance the eradicate rate of *H*. *pylori*. Certainly, there are some limits for present study and some important experiments are not performed. It would be better if inflammatory responses can be measured in the gastric tissues (for the local inflammatory responses) and within the spleen of infected mice. Furthermore, the functions of many constituents of *P*. *capitatum* were not explored. All the work will be performed in the future. Flavonoid glycosides show effects on many cytokines, which plays a critical role for the therapy of *H*. *pylori* infection. Flavonoid glycoside can affect the levels of IFN-gamma, IL-4 and gastrin but not somatostatin (Fig 8), which is also the basis for further studying the mechanisms for the functions of flavonoid glycoside.

We demonstrated that the protective and anti-bacterial functions of flavonoid glycoside from *P*. *capitatum* for the therapy of *H*. *pylori*-infected diseases. The function may be associated with its protective functions of gastric mucosa, antioxidant bioactivities and regulation for the levels of IFN-gamma, IL-4, gastrin and somatostatin. All these functions can reduce the injury of gastric tissues infected by *H*. *pylori* and improve the symptoms. We therefore propose that flavonoid glycoside from *P*. *capitatum* is potential for the *H*. *pylori* infection and should be developed a new drug for *H*. *pylori* infected diseases.

## Materials and Methods

### Materials and Reagent

*H*. *pylori* SS1 were purchased from the Chinese Center for Disease Control (Beijing, China). The strains were cultured on selective agar (Wilkins—Chalgren agar supplemented with 5 percent of horse blood, 10.5 ug/mL vancomycin, 0.5 ug/mL cefsulodin, 1 ug/mL trimethoprim lactate, and 1 ug/mL fungizone (Biogerm, Maia, Portugal)) and incubated at 37°C under microaerobic conditions for one day. The extracts of *Polygonum capitatum*, flavonoid glycosides were prepared according to a previous report[21, 58] and identified by Professor Ma Lin of the Institute of Material Medicine, Chinese Academy of Chinese Medical Sciences (Beijing, China). Amoxicillin, omeprazole and clarithromycin was purchased from Xinya Co., (Shanghai, China).

### Minimum inhibitory concentration (MIC) test

The determination of MICs of flavonoid glycosides for the *H*. *pylori* was examined by use of the serial dilution method as described previously [59]. Briefly, the bacteria were sub-cultured on Mueller-Hinton agar supplemented with 5 percent sheep blood for two days. A bacterial suspension with 10^7^ CFU/ml was placed onto each flavonoid glycoside dilution agar plate. After incubation for three days, the MIC of each sample was determined. Quality control was performed with *H*. *pylori* SS1.

### Lymphocyte proliferation assay

Lymphocytes were isolated from mice spleens using Amaxa Mouse T Cell Nucleofector Kit (Amaxa, Gaithersburg, USA). Lymphocyte proliferations upon the stimulation of flavonoid glycoside and controls were determined with the colorimetric Cell Counting Kit-8 (CCK-8, Beyotime, Shanghai, China). Isolated lymphocytes were plated in flat-bottom 96-well microtitre plate at a density of 5×10^5^ cells/well, 10 μl of CCK-8 was added to each well and incubated for further 4 h, and absorbing value at 450 nm was measured to count cell proliferation. The stimulation index (SI) was calculated as the ratio of mean OD value of the wells containing flavonoid glycoside-stimulated cells to mean OD value of the wells containing cells without flavonoid glycoside stimulation. All assays were conducted in triplicates.

### Model establishment

A total 100 mice C57BL/6 (6~8 weeks, male/female = 1:1, weight(20±5)g) were purchased from Experimental Animals Center of Chongqing Medical University (license No. SYXK 2007–0001). All animals were housed in cages with a 12 h light/dark cycle. The cages were kept at 23 ± 1°C with 50% relative humidity. Food and water could be available ad libitum. Animal care and handling procedures were conducted according to the International Association for Study of Pain guidelines for animals in pain research. All efforts were performed to minimize the number of animals and their suffering in the experiment. Animals were provided with sawdust bedding material and were housed under these conditions for at least 1 week prior to the experiments. Mice were fasted for 12 h before all experimental studies.

Before the infection of mice, *H*. *pylori* from plate cultures were inoculated into in Brucella broth culture medium (Becton Dickinson, Cockeysville, USA) containing 10% fetal bovine serum and were cultured for 18 h under microaerobic conditions. A total of 90 pathogen-free C57BL/6 8-week-old mice were used in compliance with guidelines and a protocol approved by the Animal Care and Use Committee of Guiyang medical college. Using a 20-gauge ballpoint metal feeding tube (Harvard Apparatus, Inc., Holliston, MA, USA), 90 mice were inoculated intragastrically with 0.1 mL of *H*. *pylori* SS1 cell suspension containing 10^8^ colony-forming units /mL on three alternate days. Ten healthy mice were inoculated with saline solution and used as a control.

After nine days, ten mice from a model group and ten from a control group were sacrificed using cervical dislocation without anesthesia prior to the end of the experiment. Subsequently, the stomachs were isolated from the mice by cutting the tissues from the esophagus to the duodenum. The non-glandular portion of fore-stomach was removed from the glandular stomach. The glandular stomach was dissected and rinsed with PBS, and divided into three longitudinal strips, which were used for bacterial culture, RNA analysis, and histology.

### Evaluation of a mouse model infected with *H*. *pylori*

Gastric tissues were homogenized via Tissue Tearor (BioSpecProducts, Bartlesville, USA). The homogenate were placed on trypticase soy agar (TSA) with different dilutions, complementing with 5 percent horse blood, 10 μg/mL nalidixic acid, 100 μg/mL vancomycin, 2 μg/mL amphotericin, and 200 μg/mL bacitracin (all antibiotics were from Sigma-Aldrich Shanghai Trading Co Ltd, Shanghai, China). After 5–7 d of culture under microaerobic conditions, *H*. *pylori* colonies were counted and the number of colony forming units per gram of tissue calculated (CFU/g). Colonies were used to test the bioactivity of urease, catalase, and oxidase. *H*. *pylori* colonies were identified using the following methods: Gram-staining, *H*. *pylori* colonies were identified using a Gram-staining kit (BD Biosciences, San Jose, USA) according to the manuscripture’s instructions; Rapid urease test, rapid urease test was performed according to a previous report[60]. A biopsy was inoculated into 1mL of 10% urea dissolved in distilled water (pH 6.8), to which two drops of one percent phenol red solution were added and incubated at 37°C for one day. A color change from yellow to pink within 1 h from the start of the test was considered a criterion for the presence of *H*. *pylori* infection; Catalase test, catalase test was conducted according to a previous report[61]. The colony grown in selective medium was placed on a slide, and bubbling following dropping 3% H_2_O_2_ was determined as positive reaction; Oxidase test, *H*. *pylori* uses disks impregnated with a reagent such as N,N,N',N'-tetramethyl-p-phenylenediamine (TMPD), which is a redox indicator[62]. The reagent can be changed from a dark-blue to maroon color when it is oxidized.

### Groups

**Fig 9:**
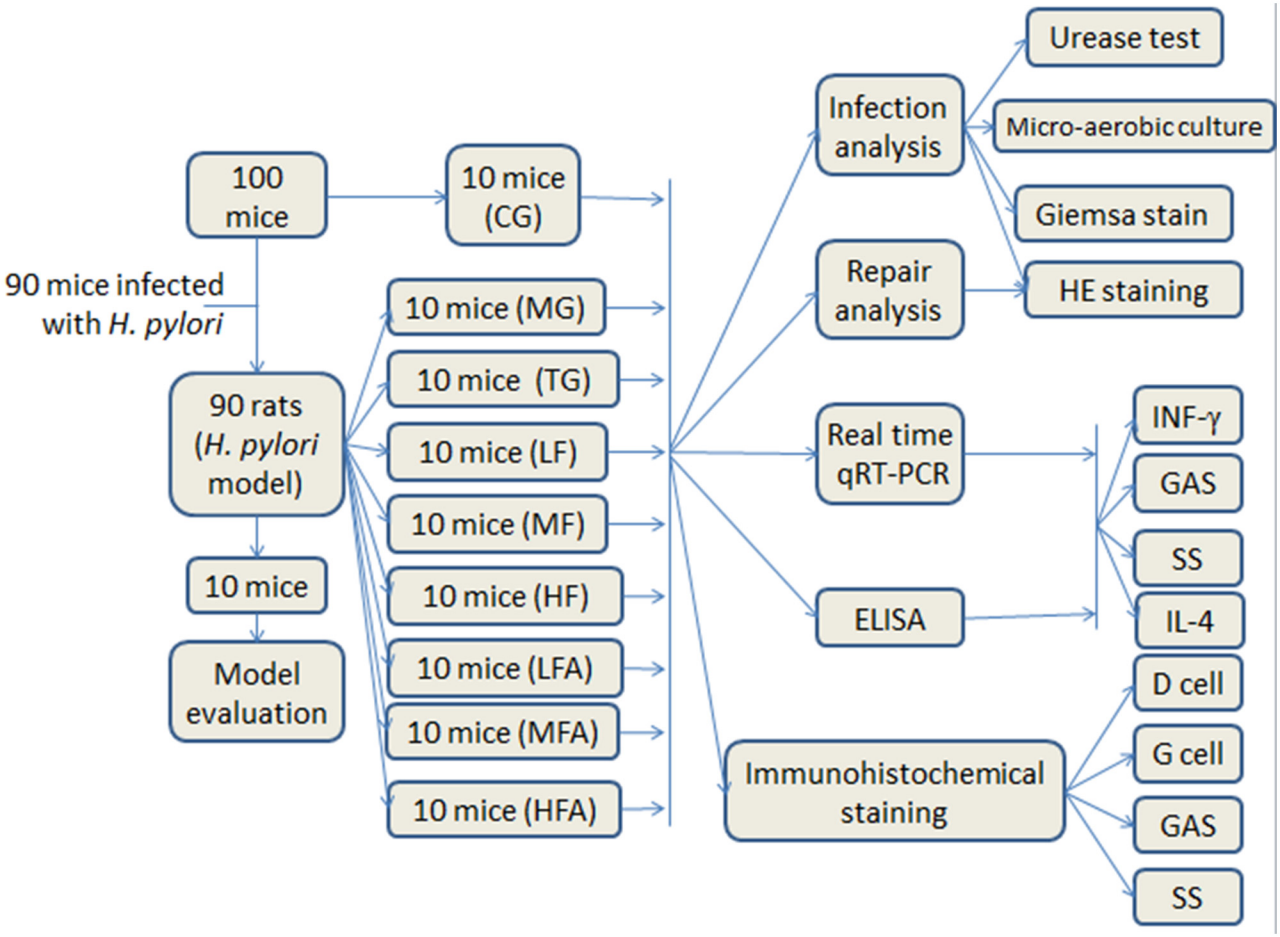
The flowchart of study. Normal mice (without *H*. *pylori* infection) were assigned as a control group (CG group) and mice models infected with *H*. *pylori* were divided into model group (MG group, only treated with saline solution), triple combination therapy group (TG group, the daily medicine intake is 0.5 ug clarithromycin, 0.02 ug omeprazole, and 1 ug amoxicillin), low/middle/high concentrations of flavonoid glycoside group (LF/MF/HF group, treated with one daily dose of flavonoid glycoside at 32/64/128 ug), low/middle/high concentrations of flavonoid glycoside and common concentration of amoxicillin group (LFA/MFA/HFA group, treated with one daily dose of Flavonoid glycoside at 32/64/128 ug and amoxicillin at 1 ug). Gastrin, GAS; Somatostatin, SS.

As Fig 9 showed, 90 mice were used for the establishment of mice models infected with *H*. *pylori* and 10 normal mice were used as control group (CG). From the mice models, 10 mice were used for the assessment of model and remaining mice were randomly divided into 8 groups. As Fig 9 showed, normal mice (pathogen free) were assigned as a control group (CG group) and mice models infected with *H*. *pylori* were divided into model group (MG group), triple combination therapy group (TG group. According to a previous report, for an adult/50 kg, daily medicine intake is 1000 mg clarithromycin, 40 mg omeprazole, and 2000 mg amoxicillin for the eradication of *H*. *pylori*[63]. For a mouse/25 g, the daily medicine intake was 0.5 ug clarithromycin, 0.02 ug omeprazole, and 1 ug amoxicillin), low concentration of flavonoid glycoside group (LF group, treated with one daily dose of flavonoid glycoside at 32 ug), middle concentration of flavonoid glycoside group (MF group, treated with one daily dose of flavonoid glycoside at 64 ug), high concentration of flavonoid glycoside group (HF group, treated with one daily dose of flavonoid glycoside at 128 ug), low concentration of flavonoid glycoside and amoxicillin group (LFA group, treated with one daily dose of flavonoid glycoside at 32 ug and amoxicillin at 1 ug), middle concentration of flavonoid glycoside and amoxicillin group (MFA group, treated with one daily dose of flavonoid glycoside at 64 ug and amoxicillin at 1 ug) and high concentration of flavonoid glycoside and amoxicillin group (LFA group, treated with one daily dose of flavonoid glycoside at 128 ug and amoxicillin at 1 ug). Mice in CG and MG groups were fed with saline solution. After two weeks, all mice were sacrificed using cervical dislocation without anesthesia prior to the end of the experiment. Each sample was fixed in 10 percent neutral formalin. The remains of tissues were stored at -80°C All the protocols for mice studies were approved by the Animal Care and Use Committees of Guiyang Medical College (Guiyang, China). The therapeutic efficiency of these groups was assessed from two aspects: 1) eradication rate of H. pylori; 2) the analysis of histochemistry and histopathology.

### Eradication rate of *H*. *pylori*

The eradication rate of H. pylori in different groups was performed according to a previous report[64].

### Analysis of histochemistry and histopathology

Hematoxylin and eosin (H&E) staining. A longitudinal strip from the greater curvature of the stomach was excised and placed in 10% normal buffered formalin for 24 h, embedded in paraffin and stained with for H&E staining according to a previous report[65]. Pathological scores of gastric tissues of the mice were graded according to the criteria described in a previous report [66].

Immunohistochemical staining for G and D cells. For each mouse, half of the stomach was totally processed for immunohistochemistry study to observe the mucosa from the distal esophagus to the duodenum. Two-micrometer-thick sections were cut from the buffered formalin-fixed paraffin-embedded tissue blocks and placed onto Super frost plus slides (Menzel-Gläser, Braunschweig, Germany). After baking in an oven, the sections were dewaxed and rehydrated. Endogenous peroxidase was blocked with 2% H_2_O_2_ in absolute methanol for 10 min. Monoclonal Mouse anti-Gastrin antibody (#G2020-08, Beijing Huamei Scientific, Beijing, China) and Monoclonal Mouse anti-somatostatin antibody (#MA5-17182, Thermo Fisher Scientific, Inc., Rockford, USA) were used and immunohistochemical staining for G and D cells was conducted with the strept-avidin-biotin-peroxidase complex (Byotime, Shanghai, China). Five images were randomly viewed under microscope (200×) from each anti-gastrin immunohistochemical staining section. The number and grey values of G cells were counted by a computer. The number and grey values of D cells were measured in the same way as the G cells.

### Real-time qRT-PCR

RNA was isolated from the stomach using t using a RNA isolation kit (Bioteke, Beijing, China). The cDNAs were synthesized from purified RNA with Reverse Transcription Kit (Takara, Dalian, China). The mRNA levels of IFN-gamma, IL-4, Gastrin, Somatostatin and H. pylori specific gene CagA (GenBank No. AB090103.1)[42], were measured using the primers were synthesized as Table 7. For real time qRT-PCR, β-actin was used as the normalizer, and tissue from uninfected mouse stomachs served as a blank control. All cDNA samples were analyzed in triplicate, along with β-actin controls. Levels of mRNA are compared between the tissue from *H*. *pylori*-infected mice and the tissue from uninfected mice. Relative units were calculated as 2-^ΔΔ^Ct (Ct, cycle threshold) where ^ΔΔ^Ct is equal to the difference between the ^Δ^Ct of the gene of interest of the experimental sample and the calibrator tissue. The ^Δ^Ct of target genes were calculated as the difference between the cycle threshold of target genes and the cycle threshold of β-actin.

**Table 7:** Primers used in real-time qRT-PCR.

Genes	Primers (5' to 3')	Size(bp)
**β-actin A0009**	GAGACCTTCAACACCCCAGC	263
**β-actin A0010**	ATGTCACGCACGATTTCCC	
**CagA F1**	ttcagtaaggtagagcaagc	180
**CagA R1**	caattctttcctgatatccg	
**GAS F1**	TGCTGGCTCTAGCTACCTTCTC	230
**GAS R1**	TCCGTAGGCCTCTTCTTCTTC	
**SS F1**	GAGCCCAACCAGACAGAGAAT	151
**SS R1**	AGAAGTTCTTGCAGCCAGCTT	
***IFN-gamma* F1**	TGGCTGTTTCTGGCTGTTACT	218
***IFN-gamma* R1**	GATGGCCTGATTGTCTTTCAA	
***IL-4* F1**	GTCCTCACAGCAACGAAGAAC	241
***IL-4* R1**	TGATGCTCTTTAGGCTTTCCA	

PCR amplification was performed with an initial denaturation cycle at 95°C for 5 min, followed by 50 amplification cycles consisting of 95°C for 5 sec, annealing at 60°C for 10 sec, and extension at 72°C for 20 sec. After amplification, a melting step was performed, consisting of 95°C for 5 sec, cooling to 45°C for 30 sec (with a temperature transition rate of 20°C per second), and finally a slow rise in the temperature to 85°C at a rate of 0.1°C per second with a fluorescence decline.

### ELISA

Serum sample was placed in each well with the same volume in Microtiter plates and ELISA was performed by using ELISA Kit for according to an instruction manual (Mouse IL-4 ELISA Kit, #RAB0300, Sigma-Aldrich Shanghai Trading Co Ltd., Shanghai, China; Mouse IFN-gamma ELISA Kit, #EM1001, Pierce Biotechnology, Inc., Chicago, USA; Mouse Gastrin ELISA Kit, # CSB-E12924m, Wuhan Hi-tech Medical Devices Park, Wuhan, China; Mouse Somatostatin Elisa Kit, #CSB-E08205m, Wuhan Hi-tech Medical Devices Park, Wuhan, China). The absorption value for Nitrophenolate 158 was measured at 405 nm using Automated ELISA analyzer (Yantai Addcare Bio-Tech Co., Ltd., Yantai, China). The series of different concentrations of IFN-gamma, IL-4, Gastrin and Somatostatin were used to plot a standard curve.

### The effects of flavonoid glycosides on Gastric cells infected by *H*. *pylori*

Gastric cell line MGC803 from Shanghai Institutes for Biological Sciences, CAS (Shanghai, China). MGC803 cells were cultured in DMEM medium containing 10% fetal bovine serum (FBS) at 37°C in 5% CO^2^. *H*. *pylori* were routinely grown on sheep blood agar plates in 10% CO2 at 37°C. Before infections, *H*. *pylori* was cultured for 24 h on BAP, harvested with centrifuge, and resuspended in 1 ml of brucella broth. For biochemical assays, 5 × 10^6^ MGC803 cells were plated in 6-cm-diameter dishes with DMEM medium. The next day, the cells were washed with PBS, pH 7.0, and serum starved in serum-free DMEM for 4 h. The media were then exchanged for fresh DMEM with 2% FBS. The eukaryotic cells were then stimulated with *H*. *pylori* at a multiplicity of infection of approximately 100:1 for 1 h and cultured for one day. Subsequently, the cells were treated with a series of diluted flavonoid glycoside. After three-day culture, all the cells were washed with DMEM and lysed on ice with lysis buffer (50 mM Tris, pH 7.0, 100 mM NaCl, 1% Triton X-100, 1 mM EDTA, 1 mM EGTA). Supernatants were collected via centrifugation, and evaluated for IL-4, INF-gamma, gastrin and Somatostatin by ELISA, as described before.

### Statistical analysis

All data were analyzed via SPSS 20 software (Chicago, IL, USA). Histograms and the Kolmogorov—Smirnov methods were conducted to determine a normal distribution of the variables. With a normal distribution, quantitative data were presented as mean ± SD. T-test for independent means is used to test whether there is a difference between groups. *P* < 0.05 was regarded as statistically significant.
